# Influence of preoperative depression on clinical outcomes after cervical laminoplasty: A retrospective study

**DOI:** 10.3389/fsurg.2022.1098043

**Published:** 2023-01-09

**Authors:** Wentao Zhang, Tianze Sun, Shiyuan Wang, Jing Zhang, Ming Yang, Zhonghai Li

**Affiliations:** ^1^ Department of Orthopaedics, First Affiliated Hospital of Dalian Medical University, Dalian, China; ^2^ Key Laboratory of Molecular Mechanism for Repair and Remodeling of Orthopaedic Diseases, Dalian, China; ^3^ Department of Medical Engineering, The 967th Hospital of Chinese People's Liberation Army, Dalian, China; ^4^ Department of Orthopedics, Southwest Hospital, Army Medical University, Chongqing, China

**Keywords:** cervical spondylotic myelopathy (CSM), laminoplasty, depression, clinical outcomes, 21-item beck depression inventory (BDI), Hamilton depression scale-24 (HAMD-24)

## Abstract

**Background:**

Depression is a highly prevalent mental disorder, and we found that patients with preoperative depression had worse postoperative improvement in lumbar fusion. Are mental factors related to the prognosis of laminoplasty?

**Objective:**

To analyze the relationship between depression and clinical outcomes after laminoplasty for the treatment of multilevel CSM.

**Methods:**

In this retrospective study, 115 patients with multilevel cervical spondylotic myelopathy (CSM), who underwent laminoplasty and were followed up for more than 1 year, were enrolled in this study from October 2018 to October 2021. Patients with the scores of 21-item Beck Depression Inventory (BDI) ≥ 15 or Hamilton Depression Scale-24 (HAMD-24) > 20 were included in the depression group. The clinical outcomes were evaluated by the changes and recovery rate (RR) of Japanese Orthopaedic Association Scores (JOA) and Neck Disability Index (NDI) respectively. Univariate and multiple linear regression analyses were performed to reveal the relationship between preoperative depressive states and clinical outcomes.

**Results:**

Fourteen patients were diagnosed with depression by BDI and twenty-nine by HAMD-24. Between the depression group and the non-depression group, the age, gender, smoking history, and duration of symptoms were statistically significant (*P *< 0.05). Multiple linear regression showed that the BDI scores had a negative relationship with the changes and RR of JOA and NDI, and the HAMD-24 scores had a negative relationship with the changes and RR of JOA.

**Conclusions:**

Preoperative depression in patients with multilevel CSM can lead to worse prognosis. In order to improve the curative effect of the operation, we should pay attention to the psychological state monitoring and intervention of patients before they receive laminoplasty.

## Introduction

Cervical spondylotic myelopathy (CSM) is a disease with cervical spinal cord damage as the main manifestation due to cervical degenerative changes (1). Multilevel CSM is often accompanied by a variety of serious symptoms, and conservative treatment is often not easy to alleviate. Surgical treatment is particularly important for patients with failed conservative treatment of multilevel CSM. There are two options for the surgical approach, anterior and posterior. Anterior approach includes anterior cervical discectomy and fusion (ACDF) and anterior cervical subtotal corpectomy and fusion (ACCF), while posterior approach includes laminoplasty and laminectomy (1–3). Compared with anterior approach, posterior approach can avoid postoperative complications such as dysphagia, hoarseness, postoperative cervical instability, kyphosis, and delayed deterioration of neurological function (4–7). In posterior approach, laminoplasty is suitable for most patients, especially the elderly patients. At the same time, due to the preservation of vertebral lamina, laminoplasty can maintain the overall stability of cervical spine and reduce the incidence of postoperative complications such as axial pain and scar formation (8–10). The postoperative outcome of patients not only depends on the approach of operation, but also relates to many factors, including patient's age, duration of symptoms, neurological status and so on (11–15).

Depression is a highly prevalent mental disorder. There are about 322 million patients with depression worldwide, and the prevalence rate is about 4.4% (16). As many as 40% of people have depression symptoms at a young age, and the incidence rate of depression reaches a peak between 50 and 60 years old (17). In daily life, psychosocial and biological stressors are both the causes of depression (17, 18). At least one of the symptoms of depression must be either depressed mood or decreased positive affect (19). Depression is a common comorbidity among patients with musculoskeletal pain, and patients who are depressed complain more physical symptoms than patients who are not (20). At present, there are many screening tools for the diagnosis of depression, including the 21-item Beck Depression Inventory (BDI) (21), Hamilton Depression Scale-24 (HAMD-24) (22), the Patient Health Questionnaire-9 (23), and the Depression in the Medically ill (DMI) (24). The BDI is widely used in depression screening in view of their sensitivity, specificity and diagnostic validity (25).

In our previous study, we found that patients with preoperative depression had worse postoperative improvement in lumbar fusion (26). Are mental factors related to the prognosis of laminoplasty? In order to explore the relationship between preoperative mental disorders and postoperative effect in patients with multilevel CSM, we conducted a retrospective study to investigate the impact of preoperative depression assessed using both BDI and HAMD-24 on clinical outcomes following laminoplasty. We reviewed the literature and found that there was no study to analyze this relationship between depression and the prognosis of laminoplasty, and this study might be the first study to analyze this relationship using BDI and HAMD-24 scales.

## Materials and methods

### Patient population

This was a retrospective clinical study. This study was approved by the ethics statement of our hospital (First Affiliated Hospital of Dalian Medical University, Dalian). Between October 2018 and October 2021, 121 patients with multilevel CSM, who underwent laminoplasty took part in the study. The inclusion criteria were: (1) age ≥ 18 years old; (2) diagnosed as multilevel CSM; (3) posterior cervical approach was underwent; (4) the follow-up time was > 1 year. The exclusion criteria were: (1) previous spine surgery history; (2) postoperative surgical site infection or reoperation; (3) severe postoperative complications occurred; (4) severe mental disorder or confusion; (5) with serious underlying diseases.

### Data collection and outcome evaluations

The data collected included the patient's age, gender, body mass index (BMI), smoking history, drinking history, comorbidities (diabetes, hypertension, rheumatism), symptom duration, and surgical levels. All patients were evaluated for depressive symptoms by BDI and HAMD-24 before operation. The BDI scores ≥15 or HAMD-24 scores >20 were diagnosed as depression. BDI is widely used in clinical epidemiological investigation. It consists of 21 items, including a series of indicators such as self, environment, work, life, and future, etc. The total scores rank from 0 to 39. The higher the scores, the more serious the depressive symptoms are. BDI is a comprehensive depression assessment tool. HAMD-24 is the most widely used scale in clinical evaluation of depression, which includes 24 indexes. The HAMD-24 evaluation method is simple and the standard is clear. The total scores of HAMD-24 can reflect the severity of the disease, and can be used to evaluate the depressive symptoms of depression, bipolar disorder, neurosis and other diseases, especially for depression.

The Japanese Orthopaedic Association Scores (JOA) and Neck Disability Index (NDI) scores were used to evaluate the severity of patient's symptoms before surgery and 1 year after surgery. JOA mainly evaluates the motor function, sensation and bladder function of the limbs, the total scores are between 0 and 17, the lower the scores, the more obvious the dysfunction. The NDI scores include two parts: neck pain-related symptoms and assessment of the ability of daily living, the total scores rank from 0 to 50, higher scores predict more severe symptoms.

We used the preoperative and postoperative changes of JOA and NDI scores to indicate the patient's improvement, the changes were calculated by JOA as follows: |postoperative JOA−preoperative JOA|, by NDI scores as follows: |postoperative NDI scores−preoperative NDI scores|. The recovery rate (RR) was calculated by JOA as follows: (postoperative JOA−preoperative JOA)/(17−preoperative JOA) × 100% (27), by NDI scores as follows: (postoperative NDI scores−preoperative NDI scores)/(1−preoperative NDI scores) × 100%.

### Statistical analysis

Statistical analysis was performed with the Statistical Package for Social Sciences (v.23.0, IBM Statistics, Armonk, NY, United States). All continuous variables were expressed as mean (SD, standard deviation) and compared by analysis of one-way ANOVA test or t test. Categorical variables were expressed as the number of patients with a percentage and compared by *χ*2 test or Fisher's exact test. In the final analysis, Univariate and multiple linear regression analysis were used to analyze the relationship between BDI and HAMD-24 scores and postoperative outcomes. *P* < 0.05 was considered statistically significant.

## Results

Ultimately, a total of 121 patients underwent laminoplasty were enrolled in this study, but 6 patients were excluded, because 3 patients had surgical site infection and the other 3 had hematoma after surgery. Screened by BDI, the depression group included 14 patients, including 4 males (28.6%), the mean age of patients was 72.7 ± 2.6 years, average BMI was 25.0 ± 4.3, mean hospital stay was 14.4 ± 2.3 days. Meanwhile, 8 smokers (57.1%), 1 drinker (7.1%), 2 with diabetes (14.3%), 1 with hypertension (7.1%), 3 with rheumatism (21.4%), 14 patients with >5 years (100%) of symptoms duration, and 8 with three surgical levels (57.1%), 6 with four surgical levels (42.9%). Screened by HAMD-24, the depression group included 29 people, including 10 males (34.5%), the mean age of patients was 69.4 ± 4.5 years, average BMI was 24.9 ± 3.4, mean hospital stay was 14.6 ± 2.0 days. Meanwhile, 8 smokers (27.6%), 1 drinker (3.4%), 4 with diabetes (13.8%), 2 with hypertension (6.9%), 3 with rheumatism (10.3%), 4 patients with 3–5 years (13.8%), 25 patients with >5 years (86.2%) of symptoms duration, and 15 with three surgical levels (51.7%), 14 with four surgical levels (48.3%). Similarly, patient characteristics in the non-depression group were summarized in the same way. Patients with depression screened by BDI or HAMD undergoing laminoplasty are older in age, higher in smoking proportion and females (*P* < 0.001), and have a longer duration of symptoms (*P* < 0.001)   (Table 1).

**Table 1:** Characteristics statistics of cohort by BDI and HAMD-24.

Characteristics	BDI− (*n* = 101)	BDI + (*n* = 14)	*P*	HAMD-24− (*n* = 86)	HAMD-24+ (*n* = 29)	*P*
Age (year)	58.5 ± 5.5	72.7 ± 2.6	<0.001	57.1 ± 4.5	69.4 ± 4.5	<0.001
Gender (male), *n* (%)	75 (74.3)	4 (28.6)	0.001	69 (68.6)	10 (34.5)	<0.001
BMI (kg/m^2^)	24.9 ± 2.8	25.0 ± 4.3	0.902	25.0 ± 2.9	24.9 ± 3.4	0.992
Smoking, *n* (%)	2 (2.0)	8 (57.1)	<0.001	2 (2.3)	8 (27.6)	<0.001
Drinking, *n* (%)	5 (5.0)	1 (7.1)	0.550	5 (5.8)	1 (3.4)	0.620
Diabetes mellitus, *n* (%)	13 (12.9)	2 (14.3)	1.000	11 (12.8)	4 (13.8)	0.890
Hypertension, *n* (%)	9 (8.9)	1 (7.1)	1.000	8 (9.3)	2 (6.9)	0.691
RA, *n* (%)	6 (5.9)	3 (21.4)	0.078	6 (7.0)	3 (10.3)	0.690
Symptom duration (year), *n* (%)			<0.001			<0.001
≤3	45 (44.6)	0		45 (52.3)	0	
3–5	25 (24.8)	0		17 (19.8)	4 (13.8)	
>5	17 (16.8)	14 (100)		6 (7.0)	25 (86.2)	
Surgical levels, *n* (%)			0.500			0.706
3	48 (47.5)	8 (57.1)		41 (47.7)	15 (51.7)	
4	53 (52.5)	6 (42.9)		45 (52.3)	14 (48.3)	
Hospital day (day)	13.5 ± 2.3	14.4 ± 2.3	0.190	13.3 ± 2.3	14.6 ± 2.0	0.005

BDI, 21-item Beck Depressive Inventory; BMI, body mass index; HAMD-24, Hamilton Depression Scale-24; RA, rheumatism.

The analysis of postoperative outcomes and improvement showed that there were statistically significance in postoperative JOA, NDI scores and RR between patients with depression and patients without depression in both BDI and HAMD-24 group (*P* < 0.05). In the BDI group, patients with depression had worse postoperative results, including postoperative JOA (13.5 ± 1.8 vs. 10.0 ± 1.1, *P* < 0.001), JOA changes (6.3 ± 1.6 vs. 3.0 ± 0.8, *P* < 0.001), RR (65.2 ± 16.9 vs. 30.1 ± 7.5, *P* < 0.001), postoperative NDI scores (16.9 ± 4.8 vs. 25.1 ± 3.3, *P* < 0.001), NDI changes (18.0 ± 3.9 vs. 10.1 ± 2.3, *P* < 0.001), RR (53.6 ± 12.0 vs. 29.6 ± 7.0, *P* < 0.001). Similarly, in the HAMD-24 group, patients with depression had worse postoperative results (Table 2).

**Table 2:** Postoperative outcomes and improvement comparison in BDI and HAMD-24 groups.

Clinical Outcomes	BDI− (*n* = 101)	BDI + (*n* = 14)	*P*	HAMD-24− (*n* = 86)	HAMD-24+ (*n* = 29)	*P*
**JOA**						
Pre	7.2 ± 1.3	7.0 ± 1.0	0.541	7.4 ± 1.2	6.6 ± 1.1	0.004
Post	13.5 ± 1.8	10.0 ± 1.1	<0.001	14.1 ± 1.3	10.2 ± 1.2	<0.001
Changes	6.3 ± 1.6	3.0 ± 0.8	<0.001	6.7 ± 1.3	3.6 ± 1.0	<0.001
RR (%)	65.2 ± 16.9	30.1 ± 7.5	<0.001	69.8 ± 12.7	34.5 ± 8.6	<0.001
*P*	<0.001	<0.001		<0.001	<0.001	
**NDI**						
Pre	34.9 ± 3.3	35.2 ± 2.4	0.725	34.6 ± 3.3	35.8 ± 2.8	0.081
Post	16.9 ± 4.8	25.1 ± 3.3	<0.001	15.8 ± 4.3	23.9 ± 3.6	<0.001
Changes	18.0 ± 3.9	10.1 ± 2.3	<0.001	18.8 ± 3.7	11.9 ± 2.8	<0.001
RR (%)	53.6 ± 12.0	29.6 ± 7.0	<0.001	56.2 ± 10.9	34.3 ± 8.0	<0.001
*P*	<0.001	<0.001		<0.001	<0.001	

BDI, 21-item beck depressive inventory; changes, |post−pre|; HAMD-24, hamilton depression scale-24; JOA, japanese orthopaedic association scores; NDI, neck disability index; Pre, preoperative; Post, postoperative; RR, recovery rate.

The results of univariate linear regression analysis showed that the higher the preoperative BDI and HAMD-24 scores, the worse the postoperative clinical outcomes (*P* < 0.05). After considering confounding factors, multivariate linear regression analysis showed that the BDI scores were recognized as a negative predictor of postoperative JOA (*β* = 0.171, *P* = 0.017), JOA changes (*β* = −0.147, *P* = 0.020), RR (*β* = −1.225, *P* = 0.036), and a negative predictor of postoperative NDI scores (*β* = 0.472, *P* = 0.020), NDI changes (*β* = −0.327, *P* = 0.010) and RR (*β* = −0.919, *P* = 0.027). Besides, HAMD-24 scores were negative predictor of postoperative JOA (*β* = −0.067, *P* = 0.030), changes (*β* = −0.065, *P* = 0.017) and RR (*β* = −0.554, *P* = 0.028) (Table 3).

**Table 3:** Correlation between preoperative BDI, HAMD-24 and postoperative clinical outcomes and improvement by using regression analysis.

Clinical Outcomes	Preoperative BDI				Preoperative HAMD-24			
	Univariate Regression Analysis		Multiple Regression Analysis		Univariate Regression Analysis		Multiple Regression Analysis	
	*b*	*P*	*b*	*P*	*b*	*P*	*b*	*P*
**JOA**								
Post	−0.313	<0.001	−0.171	0.017	−0.169	<0.001	−0.067	0.030
Changes	−0.269	<0.001	−0.147	0.020	−0.147	<0.001	−0.065	0.017
RR (%)	−2.951	<0.001	−1.225	0.036	−1.607	<0.001	−0.554	0.028
**NDI**								
Post	0.759	<0.001	0.472	0.020	0.403	<0.001	0.126	0.154
Changes	−0.716	<0.001	−0.327	0.010	−0.382	<0.001	−0.092	0.099
RR (%)	−2.146	<0.001	−0.919	0.027	−1.142	<0.001	−0.236	0.191

BDI, 21-item beck depressive inventory; changes, |post−pre|; HAMD-24, hamilton depression scale-24; JOA, japanese orthopaedic association scores; NDI, neck disability index; Post, postoperative; RR, recovery rate.

### Discussion

Posterior cervical approach is a common surgical method for the treatment of multilevel CSM. Liu et al. (28) indicated that ACDF was recommended when there were no more than three cumulative segments in CSM, but when there are three or more segments involved in the vertebral bodies, laminoplasty should be selected to treat multilevel CSM in order to avoid surgical complications and reoperation related to complications. Some scholars also reported that patients with ACDF and patients with laminoplasty had similar postoperative recovery, but the incidence of reoperation and complications in ACDF group was higher than that in laminoplasty group (29). Similarly, Liu et al. (30) conducted a meta-analysis, contained 11 studies with a total of 712 patients, which showed that patients in the posterior approach group had more postoperative JOA than patients in the anterior approach group. What factors cause the worse effect of posterior cervical surgery?.

Previous studies have shown there are some factors which can affect the curative effect of posterior cervical surgery, such as age (31), gender (32), smoking (33), symptom duration (34) and so on (35). Other studies have confirmed that mental factors affect the results of surgery in heart surgery, brain surgery, transplantation surgery and thoracic surgery (14, 36–40). In spinal surgery, there are some similar studies (41–43). Tuomainen et al. (44) conducted a 10-year prospective study that included a total of 102 patients with lumbar spinal stenosis and found that even patients with mild depressive symptoms had an increased risk of postoperative pain and disability. A retrospective study by Kevin et al. (45) on patients who underwent ACDF found that patients with depressive symptoms postoperatively had a significantly higher Nurick score than patients without depression. At present, there is no study on the relationship between depression and postoperative results of laminoplasty.

Therefore, we conducted this retrospective study where a total of 115 patients were included and followed up for more than 1 year. The result demonstrated that there was a relationship between preoperative depression and postoperative outcomes in patients with multilevel CSM, and patients with depressive symptoms had poorer symptoms improvement after laminoplasty. In addition, BDI and HAMD-24 were used to assess the mental state of patients, which could assess the patient's depressive symptoms more accurately. BDI includes a series of indicators, such as self-evaluation, environment, work, life, future, and so on, to judge depression symptoms, while HAMD evaluates individual depression symptoms through mental, weight, cognitive impairment, block, sleep disturbance, etc. BDI and HAMD cooperate and complement each other, at the same time, BMI can exclude the influence of anxiety disorder included in HAMD on the experimental results.

The baseline data analysis showed that compared with the patients without depression, the patients with depression screened by BDI and HAMD-24 were older in age, higher in female and smoking proportion, and had a longer duration of symptoms. Factors, such as aging of population, socioeconomic status, disability and cognitive impairment, widowhood, might lead to the result that patients with depression have an elder age (19). That Females in depression group are more is related to susceptibility, gender differences, and environmental factors, the conclusion is consistent with other research (46). The reason why there are more smokers in depression group may be related to the fact that smoking behavior and nicotine intake can enhance pleasure in people with lack of pleasure (47, 48). The longer the duration of symptoms, the longer the dysfunction associated with the disease bothers patients, which will lead to the incidence of depression (49, 50). In addition, HAMD-24 had screened more patients with depression than BDI, which might be because HAMD-24 also diagnosed some patients with anxiety symptoms as depression. In this study, the postoperative JOA and NDI scores of all patients were improved compared with those before operation, and the improvement of depressed patients was worse than that of non-depressed patients. Linear regression analysis showed that the higher the preoperative BDI scores, the worse the postoperative outcome, and the higher the preoperative HAMD-24, the worse the postoperative JOA recovery.

The effect of depression on clinical outcomes may be caused by the following factors. Depression is closely related to pain and will affect the prognosis of patients. Studies have shown that one suffering from physical diseases is often accompanied by mental symptoms, and long-term illnesses will increase their psychological burden (51). The main symptoms of multilevel CSM include pain and dysfunction. Long-term pain will seriously affect the patient's psychological mood and the quality of life (52). When the pain reaches moderate to severe, impaired body function and/or is difficult to treat, it has a clear correlation with depressive symptoms (53). In addition, depression can also cause somatization symptoms in patients, including pain, discomfort, neurasthenia, gastrointestinal symptoms, and somatosensory amplification (54, 55). Consequently, when multilevel CSM patients with depression receive a questionnaire after surgery, their pain scores may be relatively higher, leading to worse postoperative results.

Postoperative functional exercise is an important way to determine the prognosis of patients, which is helpful to the recovery of body function and the healing of surgical incision (56). Patients with depression are depressed, lack of confidence to overcome the disease, and resistance to rehabilitation training and exercise, which may lead to worse surgical results. Patients with depression also have different degrees of cognitive impairment, which may lead to a lack of correct and rational understanding of the disease and postoperative treatment, and also lead to worse prognosis (57). In addition, it has been confirmed that individuals with depression have a high level of inflammation marks (58). Depression is related to the changes of neuroendocrine immunity, which will lead to more inflammatory cytokines (59), thus affecting the rehabilitation process.

There are some limitations in this study. First of all, the sample size of the study is relatively small, with only 115 patients included in the study, which may be statistically biased, leading to inaccurate conclusions. Secondly, this is a retrospective study with a few factors included in the analysis. Some factors that have an impact on the clinical outcome may be overlooked, such as the lack of evaluation of the surgical quality, which will also have an important impact on the surgical outcome. Therefore, in order to clarify the relationship between psychological factors and surgical results more clearly, further prospective, multi-center and large-sample studies are needed.

## Conclusion

Laminoplasty can alleviate the symptoms of patients with multilevel CSM, but the improvement of symptoms in patients with depression after surgery is relatively worse. In order to improve the curative effect of laminoplasty, we should pay attention to the psychological state monitoring and intervention of patients before they receive laminoplasty.

## Data Availability

The raw data supporting the conclusions of this article will be made available by the authors, without undue reservation.
